# Relationship between problematic pornography consumption, sexual self-esteem, and sexual machismo in men aged 18 to 30 from Honduras

**DOI:** 10.3389/fpsyt.2026.1876379

**Published:** 2026-07-15

**Authors:** Ana Lucía Lovo, Sara Betsabe Herrera Perez, Rut Elizabeth Gómez, Raquel Mejía-Sánchez, Isis Suazo-Cervantes, Miguel Landa-Blanco

**Affiliations:** National Autonomous University of Honduras, Tegucigalpa, Honduras

**Keywords:** compulsive sexual behavior, gender roles, hostile sexism, problematic pornography consumption, sexual machismo, sexual self-esteem

## Abstract

**Objective:**

This study examined the associations among problematic pornography consumption (PPC), sexual self-esteem, and sexual machismo attitudes in men aged 18 to 30 years from Francisco Morazán, Honduras. It also tested whether sexual self-esteem statistically mediated the association between PPC and sexual machismo.

**Methods:**

A cross-sectional study was conducted with 392 men (*M* age = 23.7 years, *SD* = 3.63) recruited through online convenience and snowball sampling. Participants completed the Problematic Pornography Consumption Scale, the Sexual Self-Esteem subscale of the Sexuality Scale, and the Sexual Machismo Scale. Descriptive analyses, two-way MANOVA, follow-up ANOVAs, and mediation models were estimated. A second mediation model controlled for relationship status and sexual orientation.

**Results:**

Overall, 15.3% of participants were classified as at risk of PPC, whereas 84.7% reported normal levels. Sexual self-esteem scores were moderate, and sexual machismo attitudes were low to moderate. PPC was consistently associated with higher sexual machismo, including after controlling for relationship status and sexual orientation. PPC was also associated with lower sexual self-esteem. However, sexual self-esteem was not significantly associated with sexual machismo and did not statistically mediate the PPC–sexual machismo association.

**Conclusion:**

PPC was associated with sexual machismo attitudes among young Honduran men, but this association was not explained by sexual self-esteem. These findings suggest that other psychosocial, cultural, or gender-role mechanisms may better account for the link between PPC and sexual machismo. Given the cross-sectional design and non-probabilistic sampling, results should be interpreted as exploratory associations rather than causal evidence.

## Introduction

In the contemporary digital context, pornography consumption has increased significantly due to its immediate, anonymous, and free access, especially among young men (1, 2). It is worth noting that some authors refer to this phenomenon as Problematic Pornography Use (PPU) (3–5)​; however, for this study, the term Problematic Pornography Consumption (PPC) will be used. Although for many individuals it does not lead to adverse consequences, a subgroup has difficulty regulating it, evidenced by loss of control, persistence despite negative outcomes, and significant distress. This pattern is conceptualized as PPC (6). Evidence indicates that PPC is associated with anxiety, guilt, interpersonal conflicts, and unrealistic expectations about sexual performance and affective relationships. In men, this symptomatology is negatively associated with mental health, where PPC is interpreted as an externalizing behavior that intensifies as these symptoms increase. Likewise, among males, PPC is associated with reduced empathic tendencies, thereby negatively affecting the understanding of others’ emotions. Finally, psychological distress, impulsive sexual behavior, negative emotions, and loss of impulse control constitute core symptoms of PPC (2, 7–12). Likewise, repeated exposure to idealized representations has been linked to body dissatisfaction and insecurity regarding sexual performance, especially when consumption is problematic (13). Accordingly, frequent consumption of mainstream pornography may foster unrealistic body comparisons, as users tend to contrast their appearance with that of pornographic actors, internalizing body ideals associated with marked musculature and low body fat percentage, which reinforces unrealistic expectations about physical attractiveness (14). Additionally, research suggests that frequent exposure may affect couple relationships, increasing aggressiveness and devaluation of monogamy, as well as being associated with compulsive use patterns and neurobiological changes like those of other addictive behaviors (15).

Several studies have found associations between frequent pornography consumption and sexist attitudes, female objectification, and greater acceptance of rape myths, particularly when the content presents violence or degradation toward women. Furthermore, the endorsement of traditional masculinity ideologies has been linked to these outcomes, as various studies indicate that conformity to such ideologies is associated with higher levels of PPC (2, 9, 16–18). These representations contribute to the normalization of sexual scripts centered on male pleasure and unequal power dynamics (19, 20). From a sociocultural perspective, these dynamics are embedded within misogynistic belief systems that have historically sustained a double sexual standard, legitimizing male sexual activity while socially sanctioning equivalent female behaviors (21).

Supporting this view, sexual machismo is understood as the set of beliefs and attitudes that presuppose men’s right to control and dominate women in the realm of sexual intimacy. Thus, sexual machismo can be conceived as a dimension of misogyny centered on the sexual sphere, which holds the idea that men have the legitimacy to dispose of the female body, objectify it, and subject it to their own desires, without equitable consideration of women’s sexual desires. Among its most frequent manifestations are behaviors based on sexual selfishness, jealousy, infidelity, as well as physical and psychological aggression (22, 23).

Consistent with this framework, degrading language toward women constitutes a common form of verbal aggression in pornography and contributes to expanding repertoires of dehumanizing terms beyond the pornographic sphere (24–27). Thus, pornography can legitimize violence against women through fantasies of sexual abuse that naturalize their subordination (25, 28), and reinforce the interpretation of such subordination as natural and inevitable (27, 29). Contemporary research indicates that toxic masculine beliefs emphasizing domination and emotional toughness are linked to increased interpersonal and sexual violence. Men who hold hierarchical gender views are more likely to engage in aggressive behaviors and intimate partner violence (30, 31).

In this context, sexual self-esteem emerges as a key psychological variable for understanding sexual well-being. This construct refers to individuals’ evaluations of their own attractiveness, competence, and satisfaction in the sexual domain (32). It has been associated with better sexual functioning, greater satisfaction in intimate relationships, and increased security and enjoyment during sexual activity (33). Evidence suggests that sexual self-esteem does not depend solely on objective physical characteristics, but also on cognitive and emotional processes such as body self-acceptance, couple communication, and the internalization of sociocultural standards. In contexts marked by rigid gender norms and unequal sexual narratives, it can be affected by shame, insecurity, and pressure to meet performance expectations (34). Furthermore, it is closely linked to global self-esteem, as elevated self-worth is associated with greater satisfaction, better functioning, and healthier sexual behaviors (35).

In addition to its relevance for sexual well-being, sexual self-esteem could represent an important factor in understanding the relationship between problematic pornography consumption and sexist attitudes. Various authors have linked body dissatisfaction, identified as one of the possible consequences of exposure to pornography or explicit sexual content, to processes of comparison with body ideals, which fosters a negative perception of one’s own body and consequently may affect sexual self-esteem (36–39). From this perspective, sexual self-esteem emerges as a possible explanatory mechanism in the association between problematic pornography consumption and sexist attitudes. In this line, the literature has associated exposure to violent pornography with a decrease in sexual self-esteem, which in turn is related to greater acceptance of sexual coercion, understood as an expression of sexual machismo attitudes (40).

These dynamics become even more relevant when examined within specific sociocultural contexts. The interaction between sexual machismo, rigid masculinity norms, and pornography consumption acquires particular relevance in sociocultural contexts where gender inequalities persist (14). In Honduras, a study indicated that adverse childhood experiences and feelings of loneliness significantly contributed to PPC (41). This finding is particularly relevant given the high prevalence of ACEs in the country; an estimated 77% of young adults aged 18–24 have experienced at least one ACE, and 39% have experienced three or more (42). The broader context of gender-based violence further compounds this situation. Records from the Violence Observatory reported 385 homicides of women in 2023 and 240 in 2024, evidencing that lethal violence against women persists throughout the entire life cycle (43). This sociocultural context suggests that sexual machismo attitudes and the normalization of violence may contribute to the perpetuation of these dynamics (44).

**Figure 1:**
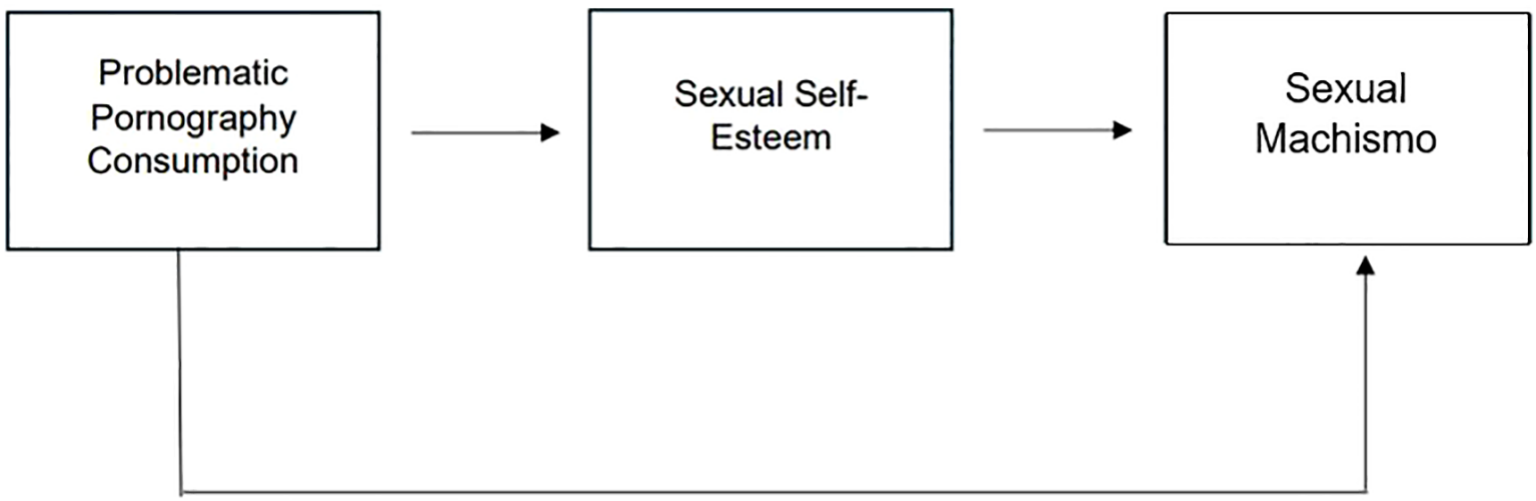
Hypothetical model.

Considering this theoretical and contextual background, the present study aimed to evaluate the direct and indirect relationship between problematic pornography consumption and sexual machismo attitudes in young men aged 18 to 30 years in Francisco Morazán, Honduras, considering the mediating role of sexual self-esteem (see **Figure 1**), as proposed in the following hypotheses:

- H1. PPC is directly and significantly associated with sexual machismo attitudes. Rationale: Previous research has examined the relationship between PPC and sexist beliefs, finding that individuals with problematic pornography consumption tend to show greater adherence to sexist ideologies (45). Likewise, PPC has been associated with processes of dehumanization toward women, which, in turn, are strongly linked to violent attitudes and behaviors against them (46). In this same line, the literature suggests a possible relationship between exposure to pornography and the manifestation of hostile sexist behaviors, as well as less egalitarian attitudes toward women (47).
- H2. Sexual self-esteem will mediate the relationship between PPC and sexual machismo attitudes. Rationale: Some authors, such as Kvalem et al. (2014) (48), have found that pornography consumption was associated with changes in men’s sexual self-esteem and body image, particularly in relation to genital self-perception. This suggests that sexual self-esteem can be affected by pornography consumption. Other authors point out that PPC has been associated with more negative evaluations of one’s own body through comparison with body ideals. On the other hand, exposure to explicit and objectifying sexual content has been linked to sexist attitudes and greater acceptance of traditional gender roles (39, 49, 50). In this context, sexual self-esteem could constitute a relevant psychological mechanism for understanding how experiences associated with PPC are connected to attitudes toward women.

In this context, the present study controlled for relationship status and sexual orientation. Relationship status was treated as a sociodemographic variable reflecting participants’ relational condition (e.g., single, married, or in a relationship), given its frequent use in well-being research and its documented association with physical and psychological health (51). Sexual orientation was also included as a control variable, as prior research suggests that it may influence pornography consumption patterns and related psychosocial variables. For example, bisexual men have been found to report higher levels of pornography consumption compared to other groups (52). Likewise, non-heterosexual men report lower levels of sexual self-esteem than heterosexual men (53). Additionally, one study has found that gay men exhibit less benevolent sexism than heterosexual men (54). These findings support the inclusion of these variables as control factors in the present study.

## Methods

### Participants

The study population consisted of men aged 18 to 30 years, residents of Francisco Morazán, Honduras, who voluntarily participated in the research by providing informed consent. Men who did not meet the established age range, resided in other departments, had not completed primary education, or did not provide informed consent were excluded from the study.

The sample size was determined based on the total population of male residents in Francisco Morazán, Honduras, which, according to data from the Population and Housing Census and departmental projections, is approximately 298,302 men (55). Because projections from the National Institute of Statistics (INE) were available for the 15–34 age group, this population was used as the closest available approximation to the target age range of 18–30 years. For the sample size calculation, a 95% confidence level and a 5% margin of error were considered. As a result, a sample size of 384 participants was established.

The sample selection process used non-probability convenience sampling, with participants selected based on their availability and accessibility. To this end, the instrument was disseminated via digital platforms, such as social media, to facilitate participation among men who met the established inclusion criteria. Likewise, participants could share the link with acquaintances who met those criteria, allowing the recruitment to expand progressively until the established sample size was reached.

The final sample comprised 392 men, exceeding the required minimum of 384. Their mean age was 23.656 years (*SD* = 3.639, Min = 18, Max = 30). Most participants identified as heterosexual (83.7%, *n* = 328), while 16.3% (*n* = 64) identified as non-heterosexual. Specifically, 6.60% (*n* = 26) self-identified as gay, 5.40% (*n* = 21) as bisexual, 2.80% (*n* = 11) with another sexual orientation, and finally 1.50% (*n* = 6) as pansexual. Because the number of participants in some specific sexual orientations was low, it was decided to group them into the non-heterosexual category for analytical purposes. Regarding relationship status, 66.1% (*n* = 259) reported being single, while 33.9% (*n* = 133) reported being in a relationship (dating, cohabiting, or married). Finally, regarding educational level, most participants (54.60%, *n* = 214) reported being currently in or having incomplete undergraduate studies. A summary of the sample characteristics is presented in **Table 1**.

**Table 1:** Sociodemographic characteristics of the sample.

Grouped sexual orientation	Frequency	% of total
Heterosexual	328	83.70%
Non-heterosexual	64	16.30%
Specific sexual orientation		
Bisexual	21	5.40%
Gay	26	6.60%
Heterosexual	328	83.70%
Other	11	2.80%
Pansexual	6	1.50%
Relationship status		
In a relationship (dating, cohabiting, or married)	133	33.90%
Single	259	66.10%
Educational level		
Incomplete secondary education	23	5.90%
Complete secondary education	64	16.30%
Ongoing or incomplete undergraduate studies (Bachelor’s or Engineering)	214	54.60%
Complete undergraduate studies (Bachelor’s or Engineering)	46	11.70%
Ongoing or incomplete postgraduate studies	33	8.40%
Complete postgraduate studies	12	3.10%

The recruitment strategy, online dissemination through social media with participant-driven chain referral, introduces systematic sampling bias that should be considered when interpreting the findings. Participants with limited internet access, lower educational attainment, or less social media engagement were less likely to be reached, resulting in a sample that was heavily concentrated among university-educated men. This educational skew may limit the extent to which the observed levels of PPC, sexual self-esteem, and sexual machismo, and the associations among them, are representative of men aged 18 to 30 in Francisco Morazán more broadly.

### Data collection techniques

A Google Forms survey format was used, with informed consent and psychoeducational resources at both the beginning and the end of the questionnaire. The data collection instrument initially included a general information section, in which participants were asked for information about their age, sexual orientation, sex assigned at birth, educational level, and relationship status.

### Problematic pornography consumption scale

The Problematic Pornography Consumption Scale (PPCS) was used in the current study (56)​. The instrument consists of 18 items that evaluate problematic pornography consumption. The PPCS items are answered using a 7-point Likert-type scale based on frequency, ranging from 1 (“never”) to 7 (“always”). Scores are obtained by summing the items, yielding a total score ranging from 18 to 126, with higher scores indicating a greater level of PPC. This study used the Spanish-adapted version of the PPCS for the adult population of Metropolitan Lima (57)​, which has been used in the Honduran setting (41). Based on the present sample data, the PPCS demonstrated adequate internal consistency (Ω = .947).

### The sexuality scale

The Sexuality Scale (SS), developed by Snell & Papini (1989) (32), assesses affective and cognitive aspects of sexuality related to personal and relational well-being. The Colombian translation of the SS was used in this study (58). For this study, only the sexual self-esteem subscale was used, which comprises 10 items with a 5-point Likert response format ranging from 1 (“disagree”) to 5 (“agree”). Scores are obtained by summing the items, taking into account the reverse scoring of negatively worded items, with a total score range from 10 to 50. After reverse-scoring negatively worded items, higher total scores indicate greater sexual self-esteem. The subscale demonstrated adequate internal consistency (*Ω* = .849).

### Sexual machismo scale (EMS-Sexismo-12)

The Sexual Machismo Scale (EMS-Sexismo-12) assesses the level of machismo or sexual sexism present in men and women, understood as a set of beliefs, attitudes, and behaviors that reflect male superiority and may lead to sexual health risk practices. The instrument was developed by Díaz Rodríguez et al. (2013) (59)​, to measure machismo from a specifically sexual perspective. The scale originally consists of 12 items, each rated on a 5-point Likert-type scale ranging from 1 (“totally disagree”) to 5 (“totally agree”). Scores are obtained by summing the items, with a total range of 12 to 60, where higher scores indicate greater sexual machismo. Based on the present sample, the EMS-Sexismo-12 demonstrated adequate internal consistency (Ω = .871).

### Data analysis

Before data collection, the research team reviewed the instruments to identify wording that could be unclear, unfamiliar, or culturally inappropriate for Honduran participants. This review focused on linguistic clarity and contextual suitability, not on modifying the underlying constructs. No substantive changes were made to item content, response formats, or scoring procedures.

Once data collection was completed, data that did not meet the inclusion criteria were removed. Then, sum scores were obtained for each scale. In the specific case of PPC, scores were classified as indicating risk of problematic consumption using a cutoff of 76 or higher, in line with previous literature (56). The data were exported to Jamovi 2.6.44 (60), where an analysis of the internal consistency of each scale was performed using McDonald’s omega (Ω).

A description of the sample was then conducted based on its demographic characteristics, using mean (*M*), standard deviation (*SD*), and absolute (*n*) and relative (%) frequencies. After this, each variable was described at the item and total score level using mean and standard deviation. A two-way MANOVA compared PPC, sexual machismo, and sexual self-esteem across relationship status (single vs. in a relationship) and sexual orientation (heterosexual vs. non-heterosexual). MANOVA was selected over separate ANOVAs because the three outcomes are intercorrelated (*r*s = −.47 to.46, all *p*s <.001), and separate univariate testing would inflate the experiment-wise Type I error rate; Pillai’s trace (*V*) is reported as the multivariate effect size. The full factorial model was estimated first to evaluate the multivariate interaction; as it was non-significant, main effects were interpreted from the additive model. Follow-up two-way ANOVAs controlled for the other grouping factor in each model, with Bonferroni-adjusted *p*-values (*p*adj = *p*raw × 3, interpreted against *α* = .05) and partial η²p as the effect size index. Homogeneity of variance was confirmed via Levene’s test.

After performing the preliminary analyses, a mediation model was evaluated to analyze the relationship between PPC and sexual machismo, considering sexual self-esteem as a mediating variable. Thus, direct, indirect, and total associations were identified at the component level. Subsequently, a second model was estimated, including relationship status and sexual orientation as covariates. Model explained variance (*R²*), estimators (*β*), and significance levels are reported. All hypothesis tests were conducted at the 95% confidence level. Mediation confidence intervals were calculated using bootstrap percentiles based on 5,000 replications. To quantify the practical significance of the mediation models, Cohen’s *f*² was computed for each regression equation using two forms: *f*² = *R*²/(1 − *R*²) for overall model fit, and *f*² = (*R*²full − *R*²reduced)/(1 − *R*²full) for the incremental contribution of specific independent variables above the remaining covariates. These values reflect local effect sizes within each regression equation, not an effect size for the indirect pathway itself. Raw *R*² values were used throughout. Conventional benchmarks were applied: *f*² = .02 (small),.15 (medium), and.35 (large) (61).

Given the cross-sectional observational design, all analyses were interpreted as tests of group differences and associations rather than causal effects. Effect-size indices were reported to quantify the magnitude of observed statistical associations or mean differences, not to imply causal influence.

### Ethical considerations

The study was approved on February 19, 2026, under code 202601007, endorsed by the Initial Integral Practice Commission of the School of Psychological Sciences at the National Autonomous University of Honduras. Confidentiality and academic use of the data were guaranteed. The data were analyzed collectively, stored securely, and anonymized, and access was restricted to the research team. Informed consent was presented digitally via Google Forms and included information on objectives, procedures, risks, voluntariness, and access to support, with the option to withdraw at any time. No physical risks were anticipated; however, a minimal psychological risk was considered, so support resources were provided at the beginning and end of the form, and no compensation for participation was offered.

## Results

### Description of scores on the problematic pornography consumption scale

On the PPCS, the mean was 48.906 (*SD* = 24.483), with scores ranging from 18 to 126. Following the classification based on the cutoff points of Bőthe et al. (2018) (56)​, of the 392 surveyed men, 15.3% (*n* = 60) were at risk of PPC, while the majority (84.7%, *n* = 332) reported normal PPC risk.

According to the data collected from the 392 respondents, the frequency of those who consume pornography is distributed as follows: “Daily or almost daily” (14.0%; *n* = 55), “1–2 times per week” (23.7%; *n* = 93), “3–5 times per week” (14.5%; *n* = 57), “1–2 times per month” (28.3%; *n* = 111), “Never” (19.4%; *n* = 76) (see **Table 2**).

**Table 2:** Frequency distribution of pornography consumption in the sample.

Frequency of consumption	Frequency	% of total
Daily or almost daily	55	14.00%
1–2 times per week	93	23.70%
3–5 times per week	57	14.50%
1–2 times per month	111	28.30%
Never	76	19.40%

The items with the highest scores were: “I have released my tension by watching pornography” (*M* = 3.416; *SD* = 1.971), followed by “When I have promised not to watch pornography anymore, I have only been able to keep it for a short period of time” (*M* = 3.212; *SD* = 2.040), and ‘I have used pornography to calm myself down’ (*M* = 3.122; *SD* = 1.892). In contrast, the items with the lowest scores were: “I have felt stressed when something has prevented me from watching pornography” (*M* = 2.288; *SD* = 1.811), followed by “I have felt that I need more and more pornography to satisfy my needs” (*M* = 2.296; *SD* = 1.686), and then “I have felt anxious when I have not been able to watch pornography” (*M* = 2.339; *SD* = 1.755). All items had a minimum of 1 and a maximum of 7.

### Description of scores on the sexual self-esteem subscale

On the sexual self-esteem scale, the mean was 35.245 (*SD* = 8.307), with scores ranging from 10 to 50. The items with the highest scores were: “I trust myself as a sexual partner” (*M* = 3.776; *SD* = 1.272), followed by “I am a good sexual partner” (*M* = 3.671; *SD* = 1.202), and third, “I consider myself a very good sexual partner” (*M* = 3.663; *SD* = 1.203). In contrast, the items with the lowest scores were: “I would rate myself low as a sexual partner” (*M* = 2.401; *SD* = 1.334), followed by “I sometimes doubt my sexual competence” (*M* = 2.464; *SD* = 1.339), and finally “I do not have much confidence in sexual encounters” (*M* = 2.531; *SD* = 1.355). All items had a minimum of 1 and a maximum of 5, and the total scale ranged from 10 to 50 points.

### Description of scores on the EMS-Sexismo-12

On the sexual machismo scale, the mean was 25.467 (SD = 9.702), with scores ranging from 12 to 60. The items with the highest scores were: “A man should start his sexual life in adolescence” (*M* = 2.630; *SD* = 1.228), followed by “That a man has his first sexual relationship with someone who is not his partner” (*M* = 2.582; *SD* = 1.383), and “That it is the woman who takes care of contraception for sexual intercourse or to avoid having children” (*M* = 2.365; *SD* = 1.284). In contrast, the items with the lowest scores were: “That a married man or a man in a stable relationship has sexual relations with prostitutes” (*M* = 1.760; *SD* = 1.242), followed by “A woman must accept her partner’s infidelities” (*M* = 1.821; *SD* = 1.287), and “That a man has several sexual partners at the same time” (*M* = 1.918; *SD* = 1.257). .

### Comparison of scores by relationship status and sexual orientation

The multivariate Relationship Status × Sexual Orientation interaction was tested first using the full factorial model and was not significant, *V* = .016, *F*(3, 386) = 2.12, *p* = .098; main effects were therefore interpreted from the additive model. The multivariate association with relationship status was not significant, *V* = .018, *F*(3, 387) = 2.38, *p* = .069. In contrast, sexual orientation was associated with a statistically significant but small multivariate difference, *V* = .023, *F*(3, 387) = 3.04, *p* = .029, indicating that the combined outcome profile differed across orientation groups.

Follow-up two-way ANOVAs were conducted separately for each outcome, with the other grouping factor included in each model. For sexual orientation, none of the individual outcomes reached significance after Bonferroni correction: PPC, *F*(1, 389) = 3.54, *p*adj = .182, η²p = .009; sexual machismo, *F*(1, 389) = 0.97, *p*adj = .974, η²p = .003; and sexual self-esteem, *F*(1, 389) = 3.52, *p*adj = .184, η²p = .009. The significant multivariate association with sexual orientation, therefore, appears to reflect a diffuse pattern of small correlated differences rather than a difference concentrated in any single outcome.

For the relationship status, the multivariate omnibus test was not significant; therefore, the univariate results are interpreted with caution. Sexual self-esteem was the only outcome showing a significant adjusted difference, *F*(1, 389) = 6.55, *p*adj = .033, η²p = .017: participants in a relationship reported higher sexual self-esteem (*M* = 36.80, *SD* = 8.19) than single participants (*M* = 34.44, *SD* = 8.27). PPC and sexual machismo did not differ by relationship status after correction. No Relationship Status × Sexual Orientation interaction reached significance after Bonferroni correction; the largest interaction pattern was observed for sexual machismo, *F*(1, 388) = 5.58, *p*adj = .056, η²p = .014, but this did not survive correction and is treated as descriptive (**Table 3**).

**Table 3:** Comparison of PPC, sexual self-esteem, and sexual machismo by sexual orientation and relationship status.

Relationship status	Single *M (SD)*	In a relationship *M (SD)*	*F(1, 389)*	*padj*	*η²p*
PPC	49.46 (24.56)	47.83 (24.38)	0.25	>.999	.001
Sexual Machismo	25.47 (9.56)	25.46 (10.01)	0.01	>.999	<.001
Sexual Self-Esteem	34.44 (8.27)	36.80 (8.19)	6.55	**.033**	.017
Sexual orientation	Heterosexual *M (SD)*	Non-heterosexual *M (SD)*	*F(1, 389)*	*padj*	*η²p*
PPC	47.86 (24.43)	54.27 (24.23)	3.54	.182	.009
Sexual Machismo	25.68 (9.80)	24.38 (9.17)	0.97	.974	.003
Sexual Self-Esteem	35.62 (8.06)	33.31 (9.31)	3.52	.184	.009
Relationship status × sexual orientation	*—*	*—*	*F(1, 388)*	*padj*	*η²p*
PPC	—	—	0.22	>.999	<.001
Sexual Machismo	—	—	5.58	.056	.014
Sexual Self-Esteem	—	—	0.08	>.999	<.001

Main-effect follow-up ANOVAs were estimated from additive two-way models including relationship status and sexual orientation. Interaction effects were estimated from full factorial models. Means and standard deviations are unadjusted descriptive statistics. Bonferroni-adjusted *p* values are reported across the three outcomes (*p*adj = *p*raw × 3). ηp², partial eta squared. Dashes indicate that marginal means are not displayed for interaction tests. Significant adjusted *p*-values are presented in bold.

### Mediation analysis

A mediation analysis was conducted to identify the direct and indirect relationship between PPC and sexual machismo, considering the mediating role of sexual self-esteem. The model was statistically significant and explained 20.90% of the variance in sexual machismo scores, *F* (2, 389) = 51.516, *p* <.001. At the direct level, there was a statistically significant relationship between PPC and sexual machismo (*β* = 0.467, *p* <.001), indicating that higher PPC is associated with higher sexual machismo scores. The analysis of the indirect relationship indicated that it is not mediated by sexual self-esteem (*β* = -0.010, *p* = .686). At the component level, PPC was inversely related to sexual self-esteem (*β* = -0.468, *p* <.001). However, sexual self-esteem was not significantly related to sexual machismo (*β* = 0.021, *p* = 0.685). The total relationship also confirms that PPC is positively associated with sexual machismo (*β* = 0.457, *p* <.001) (see **Table 4**).

**Table 4:** Results of the mediation model.

Type	Relationship	Estimate	SE	95% CI LL	95% CI UL	β	z	p
Indirect	PPC ⇒ Sexual Self-Esteem ⇒ Sexual Machismo	-0.004	0.009	-0.025	0.016	-0.01	-0.405	0.686
Component	PPC ⇒ Sexual Self-Esteem	-0.159	0.015	-0.192	-0.127	-0.468	-10.5	**<.001**
	Sexual Self-Esteem ⇒ Sexual Machismo	0.024	0.059	-0.103	0.15	0.021	0.405	0.685
Direct	PPC ⇒ Sexual Machismo	0.185	0.02	0.141	0.23	0.467	9.185	**<.001**
Total	PPC ⇒ Sexual Machismo	0.181	0.018	0.142	0.222	0.457	10.17	**<.001**

Confidence intervals computed with method: Bootstrap percentiles with 5,000 replications. Significant *p*-values are presented in bold. PPC, Problematic Pornography Consumption.

To complement the analyses, a second mediation model was implemented in which relationship status and sexual orientation were controlled for. This model obtained an R² of 0.218, equivalent to 21.80% of the explained variance in sexual machismo. This represents a marginal improvement of 0.90% over the original model, *F* (4, 387) = 27.000, *p* <.001. At the direct level, sexual machismo scores were related to PPC (*β* = 0.472, *p* <.001) and sexual orientation (*β* = -0.094, *p* = .037), but not with relationship status (*β* = -0.006, *p* = .891). Furthermore, sexual self-esteem was not a significant mediator in the relationship between the independent variables and sexual machismo (*p* >.05). At the component level, sexual self-esteem was significantly associated with PPC (*β* = -0.460, *p* <.001) and with relationship status (*β* = -0.117, *p* = .008). Considering the total relationship, sexual machismo was associated with PPC (*β* = 0.467, *p* <.001) and with sexual orientation (*β* = -0.094, *p* = .036). Thus, when comparing the original model with Model 2, the relationship among the studied variables remains, even after controlling for relationship status and sexual orientation (see **Table 5**).

**Table 5:** Results of the mediation model by relationship status and sexual orientation.

Type	Relationship	Estimate	SE	95% CI LL	95% CI UL	β	z	p
Indirect	PPC ⇒ Sexual Self-Esteem ⇒ Sexual Machismo	-0.002	0.009	-0.024	0.017	-0.006	-0.249	0.803
	Relationship Status ⇒ Sexual Self-Esteem ⇒ Sexual Machismo	-0.03	0.123	-0.327	0.256	-0.001	-0.248	0.804
	Sexual Orientation ⇒ Sexual Self-Esteem ⇒ Sexual Machismo	-0.017	0.069	-0.252	0.183	-0.001	-0.243	0.808
Component	PPC ⇒ Sexual Self-Esteem	-0.156	0.015	-0.188	-0.125	-0.46	-10.368	**<.001**
	Sexual Self-Esteem ⇒ Sexual Machismo	0.015	0.06	-0.115	0.143	0.013	0.249	0.803
	Relationship Status ⇒ Sexual Self-Esteem	-2.045	0.775	-3.515	-0.512	-0.117	-2.637	**0.008**
	Sexual Orientation ⇒ Sexual Self-Esteem	-1.13	0.998	-3.159	0.872	-0.05	-1.133	0.257
Direct	PPC ⇒ Sexual Machismo	0.187	0.02	0.142	0.232	0.472	9.325	**<.001**
	Relationship Status ⇒ Sexual Machismo	-0.126	0.925	-1.917	1.731	-0.006	-0.137	0.891
	Sexual Orientation ⇒ Sexual Machismo	-2.459	1.181	-4.705	-0.223	-0.094	-2.082	0.037
Total	PPC ⇒ Sexual Machismo	0.185	0.018	0.146	0.225	0.467	10.382	**<.001**
	Relationship Status ⇒ Sexual Machismo	-0.157	0.918	-1.906	1.643	-0.008	-0.171	0.864
	Sexual Orientation ⇒ Sexual Machismo	-2.476	1.181	-4.728	-0.253	-0.094	-2.097	**0.036**

Confidence intervals computed with method: Bootstrap percentiles with 5,000 replications. Significant *p*-values are presented in bold. PPC, Problematic Pornography Consumption. Relationship status coded 0, in a relationship; 1, single; sexual orientation coded 0, heterosexual; 1, non-heterosexual.

The sexual machismo outcome equation produced a medium overall association (*f*² = 0.26, Model 1; *f*² = 0.28, Model 2), with the explained variance concentrated almost entirely in PPC: the incremental variance uniquely associated with PPC in the sexual machismo equation was medium, approaching large (*f*² = 0.22 in both models), while sexual self-esteem accounted for negligible additional variance once PPC was included (*f*² <.001), consistent with the non-significant *b* path and the absence of an indirect association. Sexual orientation showed a very small unique association with sexual machismo (*f*² = 0.01, below Cohen’s small-effect threshold), and relationship status was negligible (*f*² <.001). In the mediator equation, PPC was associated with a medium, approaching a large proportion of variance in sexual self-esteem (Model 1: *f*² = 0.28; Model 2: *f*² = 0.27); this association was not, however, paralleled by a corresponding association between sexual self-esteem and sexual machismo. Across both models, PPC was the only variable with a practically meaningful *f*² value.

The results of the study partially support the proposed hypotheses. First, Hypothesis 1 is supported, as a significant relationship was demonstrated between PPC and sexual machismo. In contrast, Hypothesis 2 was not supported, as sexual self-esteem was not a significant mediator.

## Discussion

This study analyzed the relationship between Problematic Pornography Consumption, sexual self-esteem, and sexual machismo attitudes in 392 men aged 18 to 30 years residing in Francisco Morazán, Honduras. Regarding PPC, most participants were within the normal range; however, 15.3% were at risk, indicating that although it is not a predominant phenomenon in the sample, it constitutes a relevant group that may require attention. The highest scores were associated with the use of pornography as a strategy to release tension or to calm down, which is consistent with the literature linking PPC to the presence of psychological distress and its use as an emotional regulation mechanism (62). Regarding sociodemographic variables, no statistically significant differences were found by relationship status or sexual orientation, indicating that PPC is distributed relatively homogeneously across these groups.

With respect to sexual self-esteem, the results show moderate levels in the sample, with a mean of 35.245, suggesting average sexual self-esteem. The items with the highest scores reflect confidence and perceived efficacy as a sexual partner, while the lowest scores are associated with doubt and insecurity in sexual performance. This interpretation is consistent with studies that conceptualize sexual self-esteem as part of the sexual self-concept, reflecting perceptions of competence and efficacy in intimate situations (63).

As for sexual machismo, the results reflect relatively low to moderate levels in the sample, with a mean of 25.467. These results are consistent with studies indicating that sexist attitudes may manifest more subtly through cultural norms and traditional beliefs about sexual behavior, rather than in openly hostile expressions (64).

Regarding the mediation analysis, the results indicate that PPC is directly and significantly associated with sexual machismo, meaning that higher levels of PPC are associated with higher levels of sexual machismo attitudes. However, sexual self-esteem does not play a significant mediating role in this relationship, as no significant association was found between sexual self-esteem and sexual machismo. Nonetheless, PPC was inversely related to sexual self-esteem, suggesting that higher PPC is associated with lower perceived sexual competence. Taken together, these findings indicate that the relationship between PPC and sexual machismo is primarily direct and not explained by sexual self-esteem.

When incorporating relationship status and sexual orientation as control variables, the results remain consistent with the original model. PPC continues to be a significant correlate of sexual machismo. The magnitude of this association, assessed using Cohen’s f² statistic, was 0.22 in both models, an effect size approaching the upper limit of the medium range. This indicates that PPC and sexual machismo have a substantial relationship, which is also consistent with other studies reporting fewer egalitarian attitudes toward women associated with prior pornography consumption, as well as higher levels of hostile sexism in young men aged 18 to 35 who consumed pornography compared to those who did not. Additionally, sexual orientation also shows a significant association, unlike relationship status, which presents no relevant association (47, 64).

Sexual self-esteem still does not play a significant mediating role. This could be explained by the fact that sexual self-esteem reflects individual perceptions and intrinsic aspects, such as evaluations of genital satisfaction, personal experiences, and body image, whereas sexual machismo responds to sociocultural patterns and beliefs as well as gender role norms, predominantly based on male domination over female submission (47, 48, 64, 65). Therefore, both variables operate at different levels. The role of other variables, such as personality traits, should also be considered. Some authors associate Dark Triad personality traits with the acceptance of sexual aggression myths that reflect patterns of power and control desires characteristic of pornography and explicit sexual content, as well as peer norms, since the literature indicates that the perception of acceptance within peer circles is associated with negative attitudes toward women (66, 67). This absence of mediation is also reflected in the effect sizes. This result aligns with the findings obtained, since the unique variance associated with sexual self-esteem, once PPC was included in the model, was practically null (*f² < 0.001*), well below the threshold for a small effect. This indicates that, even if a bivariate association between sexual self-esteem and sexual machismo existed, such an association disappears when controlling for PPC, reinforcing the conclusion that sexual self-esteem does not operate as a relevant explanatory mechanism in this relationship. Nevertheless, pornography consumption is associated with sexist attitudes, which may vary by gender and sexual orientation. Given that sexism constitutes an important basis for gender inequality, future studies should examine how pornography consumption interacts with these pre-existing belief systems (64).

This study is relevant in the Honduran context as it provides empirical evidence on the relationship between PPC, sexual self-esteem, and sexual machismo attitudes in young men.

Given the cross-sectional nature of this study, causal conclusions cannot be drawn. Nevertheless, these findings point to potential directions for future exploration that could inform national initiatives. Organizations such as CARE Honduras (68), which aims to reduce inequality by promoting a society free of discrimination through community-based programs such as *Libres, Seguras y Diversas* [Free, Safe, and Diverse], whose objective is to eradicate gender-based violence by addressing the social norms that perpetuate it.

Similarly, World Vision Honduras (69), an organization focused on child protection and humanitarian assistance, promotes programs such as MEN, whose objective is to foster positive masculinities by providing participants with tools and spaces to examine gender stereotypes critically (70), in which the findings of this study could provide elements for future lines of awareness-raising and educational work.

Within the field of comprehensive sexuality education, the *Secretaría de Salud* [Ministry of Health in Honduras] (SESAL) (71) has developed guidelines and educational materials such as *Cuidando mi Salud y mi Vida* [Caring for My Health and My Life], focused on sexual and reproductive health; however, the approach to problematic pornography consumption and its possible influence on the construction of sexuality remains limited. In this context, the results of the present study may be considered a preliminary input for strengthening sexuality education programs, particularly regarding the incorporation of critical approaches to digital sexual content consumption.

The study presents several limitations that should be considered when interpreting its results. First, the cross-sectional and observational design does not allow temporal ordering or causal inference. Therefore, the observed associations should not be interpreted as evidence that problematic pornography consumption produces sexual machismo attitudes or changes in sexual self-esteem. Alternative temporal sequences are plausible. For example, sexual machismo may contribute to problematic pornography consumption, rather than the reverse. Previous studies suggest that men with more hostile attitudes toward women may be more likely to consume explicit content or pornography involving violent scripts and sexual coercion (72, 73). It is also possible that both problematic pornography consumption and sexual machismo are shaped by broader factors not assessed in the present study, such as rigid gender-role beliefs, peer norms, religiosity, social desirability, sex education, substance use, or relationship experience.

Second, the sampling strategy limits the generalizability of the findings. The sample was non-probabilistic and recruited through online convenience and snowball procedures via digital media. This approach likely favored men with internet access, social media engagement, and higher educational attainment. Indeed, the sample was heavily skewed toward university-educated men, which may not reflect the broader population of men aged 18 to 30 in Francisco Morazán, particularly those with lower educational attainment, limited connectivity, or less exposure to university and online networks. Consequently, the observed levels of problematic pornography consumption, sexual self-esteem, and sexual machismo, as well as the associations among these variables, should not be generalized to young Honduran men more broadly without caution.

Third, although the instruments showed adequate internal consistency in the present sample and were reviewed for linguistic clarity and contextual suitability, some measures have not been fully validated in the Honduran context. This is especially relevant because the instruments were originally developed or adapted in other cultural settings. Therefore, potential cultural differences in the interpretation of items related to sexuality, masculinity, pornography consumption, and sexual machismo cannot be ruled out.

Fourth, due to small subgroup sizes, gay, bisexual, pansexual, and other non-heterosexual participants were grouped into a single non-heterosexual category. Although this decision improved statistical stability, it may have masked meaningful heterogeneity across sexual orientation groups. Future studies with larger and more diverse samples should examine these groups separately.

Fifth, the study relied exclusively on self-report measures. Given the sensitive nature of pornography consumption, sexual self-esteem, and sexual machismo, responses may have been affected by recall bias, social desirability, or reluctance to report stigmatized sexual attitudes and behaviors. Future studies should consider incorporating measures of social desirability or alternative data sources when feasible.

Sixth, the study did not assess the type, context, or content of pornography consumed. This is important because not all pornographic content is necessarily associated with negative outcomes. Prior evidence suggests that explicit sexual content involving aggression, coercion, objectification, or domination may be more strongly related to negative attitudes toward women than pornography consumption in general (58). Therefore, future research should distinguish between frequency of consumption, problematic consumption, content characteristics, and relational or solitary contexts of use. These distinctions would allow a more precise understanding of how pornography consumption is associated with sexual machismo attitudes among young men.

Finally, the applied directions outlined above should also be tempered by the magnitude of the present findings. Several statistically significant associations were small in effect size (e.g., *V* = .023 for sexual orientation; η²p = .017 for relationship status), and these are not a sufficient basis for targeted programmatic decisions. Only the association between PPC and sexual machismo reached a practically meaningful magnitude (*f*² approaching the medium range), so any intervention or educational implications suggested here should be anchored to that association rather than to the smaller group differences. The negligible indirect effect (*f*² <.001) further indicates that sexual self-esteem is unlikely to be a productive intervention target for reducing sexual machismo in this population, at least as a mechanism linking it to PPC. Although sexual orientation showed a small adjusted association with sexual machismo in the controlled mediation model, this finding should be interpreted cautiously because the Bonferroni-adjusted univariate comparisons did not identify significant differences in any individual outcome. Thus, this result may reflect a small model-specific association rather than a robust group difference.

For future research, it is recommended to analyze additional variables, such as emotion regulation, sexual beliefs, and masculine norms, and to broaden the sample’s diversity in sexual orientation to improve the generalizability of the findings. Likewise, it would be pertinent to explore further the psychological mechanisms arising from the relationship between PPC and sexual machismo, such as the internalization of sexual scripts, the objectification of women, and gender beliefs. Finally, it is recommended to explore the role of the type of pornographic content consumed, considering that different content characteristics could be differentially associated with attitudes toward women.

In conclusion, these findings are consistent with the possibility that problematic pornography consumption is associated with greater endorsement of beliefs that reinforce gender inequalities; however, causal direction cannot be established. From a theoretical perspective, this study expanded the understanding of the psychological factors associated with PPC and its relationship to gender beliefs among young populations. In the applied domain, the importance of incorporating these findings into the design of psychoeducational interventions, comprehensive sexuality education, and gender violence prevention is highlighted, promoting sexuality based on respect, equality, and psychological well-being. Finally, the research provided empirical evidence in a context with limited research, which allows laying the foundations for future studies on the factors influencing the development of sexual machismo attitudes and pornography consumption patterns in young populations.

## Data Availability

The raw data supporting the conclusions of this article will be made available by the authors, without undue reservation.
